# Reducing the harms of xylazine: clinical approaches, research deficits, and public health context

**DOI:** 10.1186/s12954-023-00879-7

**Published:** 2023-09-30

**Authors:** Claire M. Zagorski, Rebecca A. Hosey, Christopher Moraff, Aaron Ferguson, Mary Figgatt, Shoshana Aronowitz, Natalie E. Stahl, Lucas G. Hill, Zoe McElligott, Nabarun Dasgupta

**Affiliations:** 1 College of Pharmacy, The University of Texas at Austin, 2409 University Avenue, A1910, PHR 3.208J, Austin, TX 78712 USA; 2 HIV Prevention Research Division, Department of Psychiatry, School of Medicine, University of Pennsylvania, 3535 Market Street, Suite 4000, Philadelphia, PA 19104 USA; 3 Narcomedia, Inc., Philadelphia, PA USA; 4 National Survivors Union, 1116 Grove St, Greensboro, NC 27403 USA; 5 Department of Epidemiology, University of North Carolina Gillings School of Global Public Health, 135 Dauer Drive, Chapel Hill, NC 27599 USA; 6 University of Pennsylvania School of Nursing, Fagin Hall, 418 Curie Blvd, Philadelphia, PA 19104 USA; 7 Greater Lawrence Family Health Center, 34 Haverhill Street, Lawrence, MA 01841 USA; 8 Department of Pharmacology, Bowles Center for Alcohol Studies, University of North Carolina, CB#7178, 104 Manning Road, Chapel Hill, NC 2759 USA; 9 University of North Carolina, 725 MLK Jr. Blvd., CB 7505, Chapel Hill, NC 27599 USA

**Keywords:** Xylazine, Harm reduction, Wounds, Drug injection

## Abstract

**Objectives:**

Xylazine has emerged as a consistent part of the unregulated drug supply in recent months. We discuss major domains of xylazine’s harm, current knowledge deficits, clinical and harm reduction strategies for minimizing harm, and xylazine’s public health and policy context. As an interdisciplinary team from across the USA, we have pooled our knowledge to provide an overview of xylazine’s current and emerging contexts.

**Methods:**

To inform this essay, the pertinent literature was reviewed, clinical knowledge and protocols were shared by multiple clinicians with direct expertise, and policy and public health context were added by expert authors.

**Results:**

We describe xylazine’s major harm domains—acute poisoning, extended sedation, and wounds, along with anemia and hyperglycemia, which have been reported anecdotally but lack as clear of a connection to xylazine. Current successful practices for xylazine wound care are detailed. Understanding xylazine’s epidemiology will also require greater investment in drug checking and surveillance. Finally, approaches to community-based wound care are discussed, along with an orientation to the larger policy and public health context.

**Conclusions:**

Addressing the harms of xylazine requires interdisciplinary participation, investment in community-based harm reduction strategies, and improved drug supply surveillance. The relatively unique context of xylazine demands buy-in from public health professionals, harm reduction professionals, clinicians, basic science researchers, policymakers and more.

## Background

Xylazine was originally developed in the 1960s to treat hypertension in humans, but this application was abandoned due to excessive sedative effects [1]; today, it is routinely used as a surgical anesthetic and procedural sedative in veterinary medicine, either alone or in combination with other medications. It is supplied as an injectable solution and sold under multiple brand names. Xylazine’s primary mechanism of action is agonism of alpha-2 adrenergic receptors (α2-ARs) leading to inhibition of presynaptic norepinephrine release [2], however new data suggests there may be alternative targets [3].

**Fig. 1:**
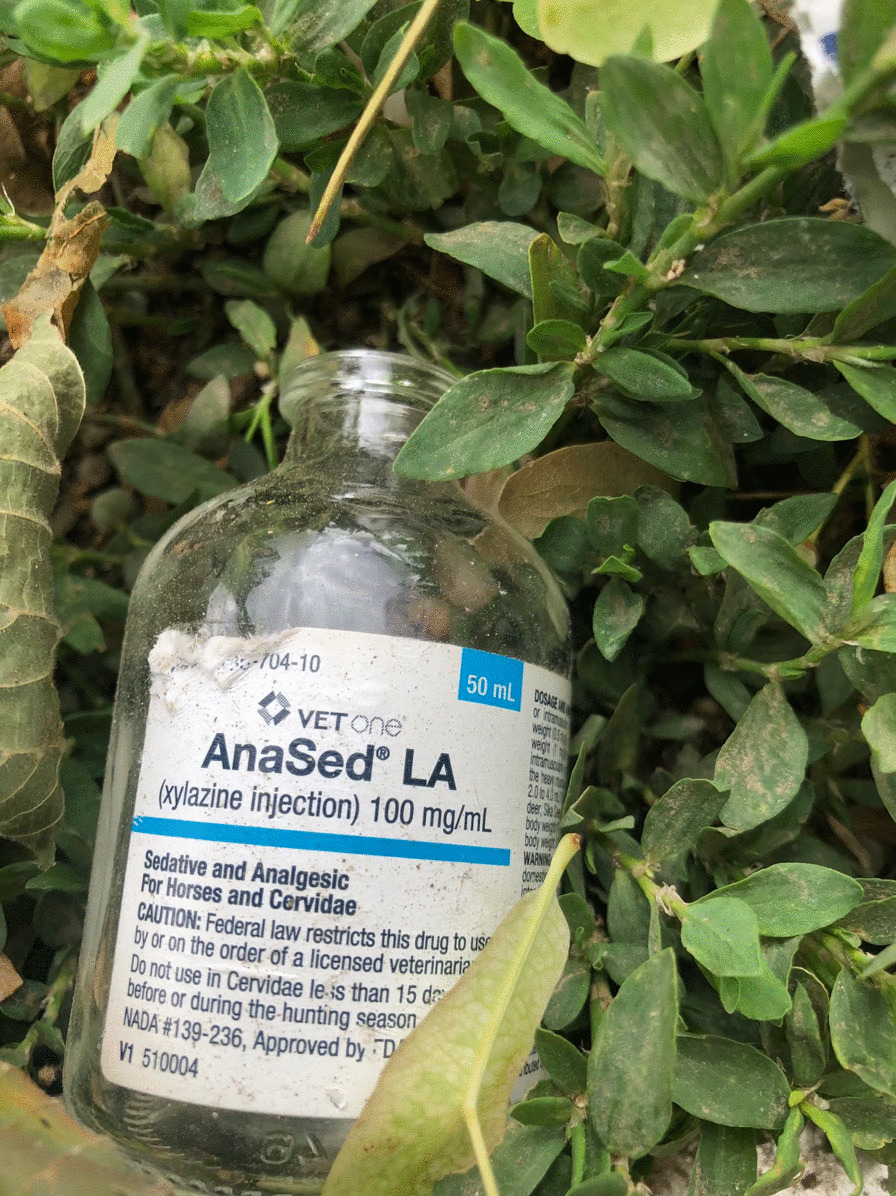
Photo of a discarded bottle of AnaSed**®** xylazine found in a shrubby area in Kensington, Philadelphia, Pennsylvania. Courtesy Christopher Moraff

**Fig. 2:**
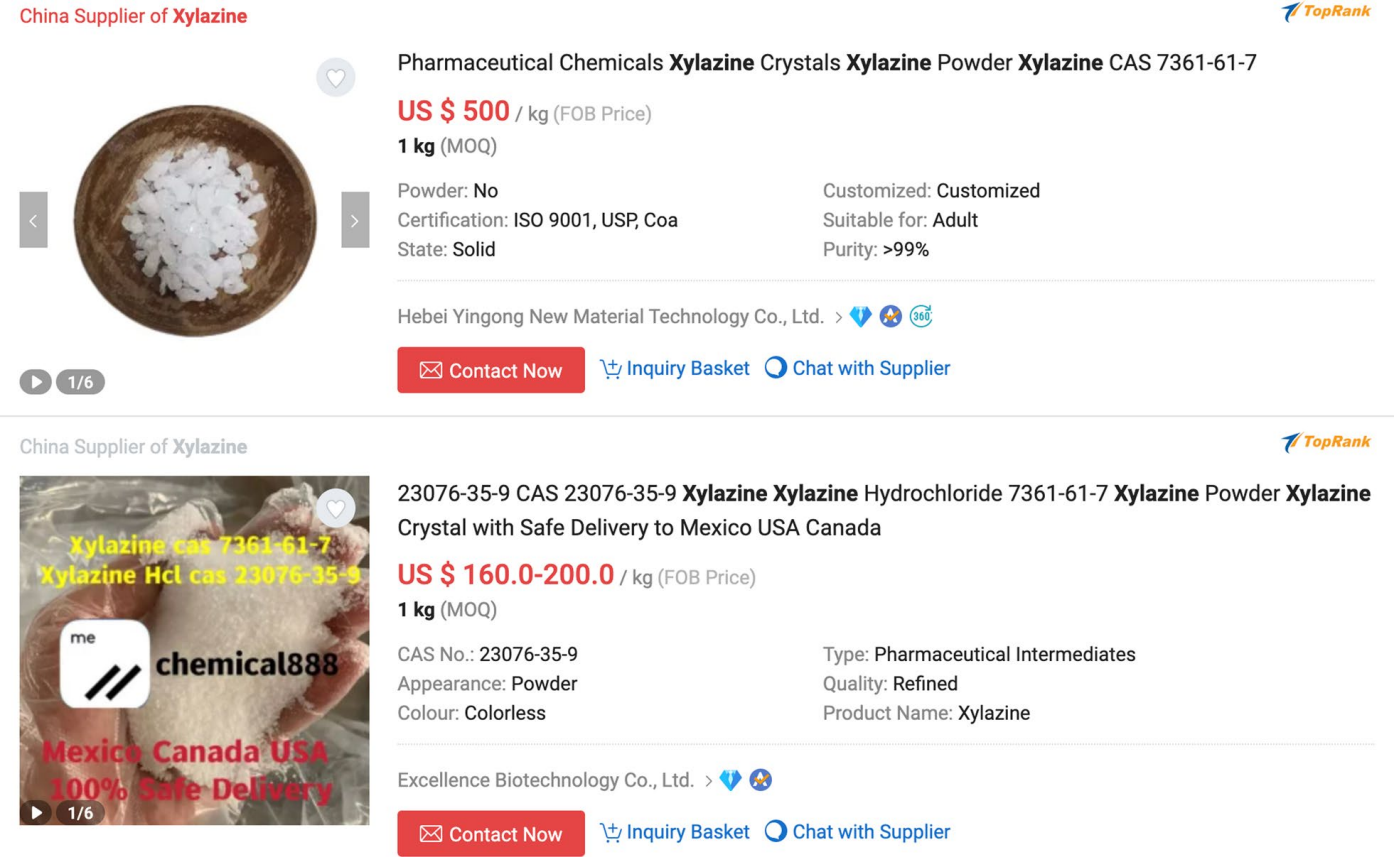
Screengrabs from an online retailer offering xylazine for purchase

Xylazine was detected intermittently in the North American unregulated drug supply for decades, and case reports of poisonings were noted as early as the 1970s and 1980s [4]. It became common in the Puerto Rican drug supply in the 2000s, where it was primarily used alongside heroin and cocaine, and where its association with skin lesions was first identified [5, 6]. Xylazine has become increasingly prevalent in the North American unregulated opioid supply in recent years, but full extent of this increase is likely under-appreciated given xylazine is not routinely assayed in post-mortem toxicology, urine drug screens, and other common modes of supply monitoring [7]. Surveillance studies are hampered because vital statistics data do not have distinct codes to identify xylazine, so xylazine would not be noted on death certificates even if detected. It is unclear whether xylazine primarily comes from the veterinary pharmaceutical supply or is synthesized clandestinely, although the presence of empty discarded vials of xylazine (specifically AnaSed**®**, Akorn Pharmaceuticals) in areas of Philadelphia known for drug sales suggests that the former is at least contributory [8]. (Fig. 1) Xylazine sold as a hydrochloride salt can be purchased on Chinese online research chemicals marketplaces, constituting another potential source (Fig. 2).

There are conflicting theories about why xylazine has been introduced into the unregulated drug supply [9]. On one hand, negative subjective feelings reported by people who use drugs about xylazine suggest incidental addition in a manner that could hamper sales. It is unclear at this point if xylazine is being sold as a pre-mixed cut, and there is likely regional variation to the specific manner in which xylazine is being processed and sold. Xylazine is found as a primary constituent in retail street samples, but also in trace quantities, further complicating whether this is from incidental inclusion or systematic addition. On the other hand, xylazine may be intentionally selected as a bulking agent given its sedative properties to allow for the inclusion of less opioid with the goal of inducing less severe respiratory depression. Given its pharmacological similarity to clonidine and lofexidine—effective treatments for opioid withdrawal syndrome—it is possible that xylazine may be intentionally selected for potential to mitigate these symptoms between opioid doses. Reports from people who use both fentanyl and xylazine support these propositions as they note xylazine increases the duration of effect from fentanyl and delays withdrawal symptoms [10]. Prior literature has demonstrated that increasing prevalence of fentanyl in the drug supply is associated with increased frequency of use [11], which is likely a response to fentanyl’s short half-life relative to that of heroin.

## Harm reduction strategies

Three primary domains of harm have emerged in relation to xylazine exposure via the unregulated drug supply—acute poisoning, prolonged sedation, and skin wounds. While there is a dearth of clinical research evaluating acute and chronic xylazine exposure in humans, collected reports from across North America reveal actionable strategies which can be employed quickly to reduce harms related to xylazine use. Given the considerable stigma people who use drugs experience in conventional healthcare settings [12, 13], identifying harm reduction strategies that empower them to self-manage symptoms and to seek support from trusted sources in their community is critical.

### Acute poisoning

Xylazine reduces level of consciousness while lowering heart rate and blood pressure. Human case reports have also identified decreased respiratory rate and the need for mechanical ventilation in instances of very high dose xylazine administration [14]. However, reports of fatalities from xylazine alone are extremely rare. From 2015–2020, the proportion of poisoning deaths in the USA involving xylazine increased substantially, but illicitly-manufactured fentanyls were present in 98.4% of xylazine-involved poisonings [15]. While xylazine may have additive or potentiating effects, this potential synergism is not yet clearly evidenced, and it is likely that fentanyls were the primary drivers of respiratory depression and death. Thus, even though naloxone does not directly and significantly antagonize the sedative effects of xylazine [3], it can nonetheless effectively treat many poisonings resulting from concomitant opioid and xylazine use, and naloxone remains a critical tool for anyone who may experience or respond to a drug poisoning. An acute reversal agent specific for xylazine has not been approved for use in humans, and whether such a drug would be helpful to reduce xylazine-associated poisoning deaths is unclear. While reversal of opioid poisoning with naloxone is generally safe even in a person with physical dependence to opioids, this is not true for all reversal agents which have been deployed in a drug poisoning context. For example, administration of flumazenil—a competitive antagonist of benzodiazepines—has resulted in death in polysubstance poisonings and induced seizures when administered to people with physical dependence to benzodiazepines [16]. Given that sudden discontinuation of other α2-AR agonists may induce rebound hypertension, it is plausible that rapid reversal of xylazine’s effects with an α2-AR receptor antagonist in a person chronically exposed to xylazine could result in a dangerous acute elevation in blood pressure [17]. Even in the absence of deployment of a specific reversal agent, clinicians must recognize that chronic exposure to xylazine may complicate withdrawal management in patients with opioid use disorder and treatment with α2-AR agonists may be warranted alongside opioid agonists [18].

### Prolonged sedation

Extended periods of sedation and immobility have been reported by people exposed to xylazine through the unregulated opioid supply [10]. While “nodding” has long been a well-known effect of opioids such as fentanyl, the sedation attributed to xylazine appears distinct in some ways. The sedation produced by xylazine is lengthier, and associated with long periods of immobility. Reports have emerged of people using xylazine and subsequently nodding in awkward positions, such as hyperflexed at the waist or slouched against a wall, for several hours. Immobility is associated with several harms that are preventable via simple and very well-studied strategies. Traditional nursing interventions to prevent harms associated with immobility (such as pressure ulcers) include padding, regular turning (every 2 h), laying people on flat surfaces free of wrinkles or other irritating prominences, avoiding skin friction while moving or turning, and ensuring that skin remains dry [19, 20]. These are all practices that laypersons can easily learn and perform. Additionally, some positions in which people may nod, such as kneeling or hyperflexion at the waist, may theoretically present a risk of distal tissue injury from arterial kinking [21] and acute compartment syndrome [22]. Moving people who have nodded in awkward positions onto their side should be encouraged.

Further, people who use drugs, harm reduction professionals, and individuals working in related fields should be vigilant for other foreseeable harms of xylazine’s prolonged sedation. For example, increased rates of sexual assault, physical assault, and theft are likely to emerge as a significant xylazine-related harm. While xylazine is too new of a drug supply component for there to be literature on this issue at this time, the context of xylazine use and prolonged sedation may be reasonably compared to that of alcohol use, which has long been known to be associated with sexual assault [23]. Additionally, particularly for people experiencing homelessness, extended periods of sedation outdoors reasonably pose a heightened risk for traumatic injury if occurring on or near streets, sidewalks, train tracks, and similar contexts.

### Skin wounds

While severe and at times limb and life-threatening skin and soft tissue infections (SSTIs) (usually attributed to *Staphylococcus ssp*. [24]) are commonly seen among people who inject drugs, the presentation of skin wounds attributed to xylazine exposure are distinct. While bacterial SSTIs are commonly seen at or near the site of injection, usually due to the introduction of bacteria by injection and associated local tissue trauma causing an abscess and/or cellulitis, one of the common hallmarks of xylazine-associated wounds is their emergence at sites other than those used for injection. These wounds are often described as large (greater than 10 cm in diameter), heterogeneous in appearance, granular and “burn-like”, and severe. An area of central necrosis is frequently observed, and a common course of progression is one of several small “punch-hole” wounds appearing in a cluster, which then coalesce into a single larger wound. Further, while wounds are often colonized with bacteria from skin/environment, they are not typically purulent, and in the absence of clear symptoms of infection do not require antibiotics. While xylazine-associated wounds are dramatic in appearance, it is important to note that they do often respond well to appropriate wound care, which should be implemented and exhausted before considering more radical options such as limb amputation. The cellular mechanisms underlying the atypical appearance and progression of these skin lesions are as-yet unknown, and basic science investigation is needed to illuminate these mechanisms.

While evidence is currently not yet sufficient to recommend standardized clinical guidelines on xylazine-related wound care the authors have a combined experience of caring for dozens of people with likely or confirmed xylazine-related skin lesions, over a period of several months, with many successes. We offer our cumulative recommendations both as a starting point for others in need of this information, and to prompt further research to help clarify and identify best practices.

Wound treatment and care for xylazine induced wounds in the outpatient setting relies on existing evidence-based practices, but must be approached with a harm reduction lens; typical or best practice treatment regimens may not be possible in the outpatient, street-based setting. Providing access to low barrier wound care with easily accessible supplies through a team-based approach (i.e., care provider and patient) may prove most beneficial. Interviewing the person with wounds in a non-judgmental, compassionate way allows for an in-depth understanding of the severity and timeline of the wound and may give way for relevant history [25]. It is important to document any previous treatments for the wound, as well as information about how the patient takes care of the wound; this interview will directly influence the type of treatments used during the visit and any upcoming appointments.

Regimens should be individualized for each patient, but can follow these major steps: 1) assess the wound and affected area, 2) cleanse wound with soap and water, wound cleanser, or saline solution depending on patient preference, product availability and potential allergy indication, 3) prepare supplies with necessary ointments or enzymatic or autolytic debriding agents (per prescribing provider order or clinic protocol), 4) dress the wound [26]. Hydrogen peroxide should not be used because it may delay wound healing via oxidative toxicity to fibroblasts [27]. In addition to these steps, motivational interviewing should be utilized to create a treatment plan in collaboration with the patient. Information about how to continue to care for the wound should be provided to the patient in a way that works for them; pre-made information sheets with symbols may be helpful, others may prefer recording the care provider's voice onto their phone with simple, replicable instructions to listen to when changing the dressing on their own.

When assessing the wound, documentation on measurements, tissue types (percent granulation, eschar, slough, presence of biofilm), signs of infection (erythema, edema, purulence) should be noted as well as systemic signs of infection (fever, chills, tachycardia) [28]. This assessment will guide the treatment course and care provision; if the wound has eschar, medical grade honey (often found as brand MediHoney® (Integra LifeSciences, Princeton, NJ)) wound gel or enzymatic collagen debridement ointments such as SANTYL (Smith + Nephew, Watford, England) may be used per protocol and indications, taking into consideration any potential allergies and contraindications [29, 30]. Mupirocin ointment, active against methicillin-resistant *Staphylococcus aureus* (MRSA), may be utilized as well [31]. These treatments, if indicated, can be applied directly to skin or a dressing such as petroleum gauze or nonadherent gauze. Before applying the impregnated gauze to the skin, care should be taken to cut the dressing to fit the wound area, while sparing any intact skin. For example, if there are areas of eschar among a greater area of slough and granulation tissue, the dressing with any enzymatic debridement should be applied to just the area indicated for treatment (eschar only), while another dressing with any indicated treatments, such as mupirocin on petroleum gauze or a nonadherent gauze, should be cut appropriately for the other types of tissue. This method prevents maceration of the wound bed while treating the most damaged tissue. Enzymatic debridements should be used with caution; it is necessary to ask the patient how often they are able to change their dressing. Enzymatic debridement will break down tissue and bacteria and if left on, can provide an ideal environment for continued bacterial growth and further infection and damage [30]. If the patient cannot change their dressing within twenty-four hours, enzymatic debridement [32] should be reconsidered. Depending on the amount of purulence, the patient's housing situation, and how long they may need to wait until their next dressing change, an ABD pad may be applied before wrapping the dressing with rolled gauze, tape, and/or elastic self-adhesive wrap.

Lesions may be present at individuals’ preferred injection locations, so education about why injection into or near lesions is harmful—in addition to general safer injection technique education—may be helpful for some patients. As venous access sites may be limited for some, a concern is a patient injecting through or alongside a xylazine-associated wound. Patients may want to inject into or near the wound in an effort to treat pain from the wound, or because prior intravenous drug use has scarred over other potential venous sites, leaving them with few options. Injecting into a wound increases the risk of bacterial superinfection of the wound and delayed healing, so alternatives to this should be explored as necessary. While non-ideal for concerns of infection and tissue injury, both subcutaneous and intramuscular injection (“skin popping” and “muscling”, respectively) are alternatives to injecting into or near a wound for people with limited points of venous access. These types of injection are also preferable to a patient accessing central veins, which carries a high risk of vessel and nerve injury [33] and systemic infection [34]. Other fruitful options include helping the patient locate new alternative points of venous access with aid of a tourniquet and/or a vein finder and encouraging use of the smallest feasible needle gauge in order to minimize local tissue injury and make use of smaller veins. Coaching the patient to drink plenty of water, apply a warm compress to the skin, and exercise the limb musculature prior to injection (e.g., performing push-ups) are also good options for increasing the intravascular volume of superficial limb veins, making these sites easier to access. Additionally, alternatives to injection altogether bypass many infection concerns. Fentanyl, which is the most-common opioid in the illegal supply at this time, may be consumed when heated on uncoated foil or in a glass pipe and the resulting vapor inhaled (“smoking”), insufflated (“snorting”) or administered rectally as a solution (“boofing”, “booty bumping”, among others). However, regional variation of the unregulated drug supply may make some routes of administration more acceptable and achievable than others, so collaboration with the patient and consideration for their experience is key. Additionally, it is not known if xylazine presents additional or more severe harms when consumed via varying routes, and so relative safety of using xylazine via a given route is unknown. As such, no specific route of administration can reasonably be described as “safer” at this time. Future research should explore the relative safety of these various routes of administration in the context of xylazine (Table 1).

**Table 1:** Xylazine harm domains and clinical approaches

Harm domain	Mechanism	Clinical implications	Harm reduction strategies
Heavy sedation	Alpha-2 receptor agonism	Continued sedation after naloxone administration Pressure ulcers and skin breakdown likely Elevated risk for DVT Elevated risk of compartment syndrome Nerve, muscle, and soft tissue injury Rhabdomyolysis	Encourage using drugs with a friend Roll people nodding onto their side Move people nodding every two hours Pad under bony areas (sacrum, heels, shoulders, etc.) Avoid wrinkled or hard surfaces under nodding person
Skin wounds	Unknown	Bacterial superinfection possible Ensure adequate longitudinal wound care Can cause shame and reduced care-seeking Individuals may be deemed ineligible for in-patient substance use disorder care due to untreated wounds	Coach to avoid injecting into or near wounds Facilitate wound care access Teach individual and friends/family how to care for wounds Provide wound care supplies Teach on signs of worsening condition
Anemia	Unknown; perhaps sympathetic antagonism	Vague symptoms may delay care-seeking Important to rule out dietary or other causes	Coach communities on signs and symptoms; when care-seeking is necessary Encourage and facilitate community support to identify changes in health status in self and others
Dysglycemia	Unknown	Vague symptoms may delay care-seeking Important to rule out other causes of metabolic derangement Potential to worsen skin wound progress	Coach communities on signs and symptoms; when care-seeking is necessary Encourage and facilitate community support to identify changes in health status in self and others

### Anemia and hyperglycemia

Transient anemia and hyperglycemia have been observed with xylazine administration in animals [35, 36]. Whether these may be observed in humans and progress to a level of severity warranting specific intervention in humans as a result of chronic exposure is unknown. Further research in this area is needed. While specific harm reduction strategies for addressing anemia and hyperglycemia are unclear, the larger-picture fundamentals of harm reduction are still very apt. Many of the early signs and symptoms of these two disease conditions—weakness, fatigue, dizziness, dry mouth—are non-specific and easily ignored. Thus, a fruitful harm reduction strategy may include providing education about signs and symptoms in case these conditions appear, supporting community cohesion among people who use drugs to encourage the observation of changes in health status in self and others, and offering to accompany potentially ill people to the hospital or clinic to advocate and help buffer against hostility and stigmatizing interactions from health care staff.

## Awareness and engagement

The relatively rapid ascendance of xylazine as a component of the illegal drug supply, and the near-total lack of informative research on xylazine in humans, speaks to an under-appreciated danger of continued and expanded drug prohibition: the acceleration of the drug market away from well-understood substances such as heroin, to less-familiar substances which are relatively unknown to people who use drugs, clinicians, researchers, and policymakers, and which pose unknown health risks. While xylazine is an excellent example of this, so too are ascendant drug supply components such as the nitazene opioids and novel benzodiazepines such as flualprazolam. We urge rapid attention to xylazine in the basic sciences to illuminate its cellular mechanisms of action, and in the clinical context to inform best practices for management of xylazine-related health harms—in the etiologic sense of preventing xylazine-related wounds, and in the larger policy and social science context. Without these insights, comprehensive and scientifically rigorous care for people who use xylazine cannot be achieved.

### Harm reduction leadership

Considering that one of xylazine’s hallmarks is severe wound formation, resources should be moved to harm reduction organizations, street outreach groups, and mutual aid organizations with close and preexisting relationships with people who use drugs in their community. For people who use drugs, a major barrier to seeking medical attention is maltreatment in the medical setting. This is particularly true for people seeking care for skin and soft tissue infections [13]. Since wounds such as those associated with xylazine use often require wound care over the course of several weeks, if this care was only offered in a traditional clinical setting it would likely fail to be broadly accepted and utilized as appropriate. Alternatively, organizations which have already built trusting relationships with people who use drugs are in an ideal position to provide necessary care, and so should be funded and empowered to do so for best results at the community level. The incorporation of wound care services into traditional harm reduction settings like syringe access programs can alleviate stigma, provide low barrier access to high quality care for participants, and help establish a point of care for individuals to receive basic treatment and share information with healthcare staff and each other [37].

### Drug checking

Drug checking is one strategy to lessen the harms of xylazine. This approach involves testing drug samples for the presence of specific substances via methodologies such as immunoassay test strips and spectroscopy. People who use drugs may be unknowingly using xylazine, particularly when its presence in the drug supply is new to a region, and given its common commingling with opioids in the drug supply. In the case of xylazine, knowing of its presence can help people who use drugs make informed decisions guided by harm reduction practices. At the population level, comprehensive drug checking data can be aggregated to understand spatiotemporal trends in xylazine prevalence. Toxicology testing is another potential source of information about xylazine trends [15]. This information can also be a complement to drug checking information, however, there are limitations in this context. Toxicology testing is typically limited in its timeliness as it often occurs post-mortem, and due to common lags in data reporting. Additionally, xylazine is not yet a standard component of toxicology panels, making reporting from this domain imprecise for population-level insights.

### Nothing about us without us

The empowerment of people who use drugs to check, disseminate, and act upon information about the unregulated drug supply is central to successfully mitigating the risks of xylazine and other contaminants. Both drug-using and selling communities (often one and the same) have the ability to rapidly communicate critical information obtained through empirical observation in an actionable and beneficial way to drug-using communities [38]. For this reason, people who use drugs must be empowered to engage in and lead self-determinant practices such as confidential drug checking, and should be employed whenever possible in testing settings [39]. Working directly with people who use drugs to form and implement drug checking and harm reduction strategies illustrates the scientific value of parsimony, and works to mitigate biases against people who use drugs. Bias against people who use drugs is a significant and omnipresent potential confounder which threatens valid conclusions in research. Through the meaningful inclusion and empowerment of people who use drugs in research relevant to this population, we confront threats to the validity of this research and create a richer and more insightful research community around drug use, while providing employment and pathways to career advancement to experts that are often excluded from the workforce owing to their drug use.

### Drug war failure

Xylazine is unscheduled at the federal level, and to date is illegal only in Florida, where it was banned in 2018 [40]. Increased attention to xylazine and its harms has recently prompted a call for xylazine to be federally scheduled. However, we urge caution in such an approach. Scheduling xylazine would only control the licit supply, creating barriers to needed research on the health impacts, toxicology, and safety profile of the drug. Further, xylazine is used very commonly as a surgical anesthetic for animals used in pre-clinical research, including cancer research. Scheduling xylazine would disrupt these research areas and slow progress. Additionally, the ban of xylazine in Florida serves as a test case of sorts, and the results are wanting. Despite making xylazine illegal in 2018, the Drug Enforcement Agency (DEA) reports a 1127% increase in xylazine-involved overdose deaths in Florida’s field division [41] from 2020 to 2021. And finally, increasing prohibitions on substances has been shown repeatedly to prompt a less-safe drug market. With greater prohibition, the supply side of the unregulated drug market is motivated to invest in more potent substances which are logistically easier to conceal [42], clouding our understanding of the safety of the drug market and moving the drug supply away from one of consistency. An inconsistent and more-potent drug supply heightens health risks to people who use drugs.

## Data Availability

Data sharing is not applicable to this article as no datasets were generated or analysed during the current study.
