# Correction: Reducing the harms of xylazine: clinical approaches, research deficits, and public health context

**DOI:** 10.1186/s12954-023-00903-w

**Published:** 2023-11-27

**Authors:** Claire M. Zagorski, Rebecca A. Hosey, Christopher Moraff, Aaron Ferguson, Mary Figgatt, Shoshana Aronowitz, Natalie E. Stahl, Lucas G. Hill, Zoe McElligott, Nabarun Dasgupta

**Affiliations:** 1 College of Pharmacy, The University of Texas at Austin, 2409 University Avenue, A1910, PHR 3.208J, Austin, TX 78712 USA; 2 HIV Prevention Research Division, Department of Psychiatry, School of Medicine, University of Pennsylvania, 3535 Market Street, Suite 4000, Philadelphia, PA 19104 USA; 3 Narcomedia, Inc., Philadelphia, PA USA; 4 National Survivors Union, 1116 Grove St, Greensboro, NC 27403 USA; 5 Department of Epidemiology, University of North Carolina Gillings School of Global Public Health, 135 Dauer Drive, Chapel Hill, NC 27599 USA; 6 University of Pennsylvania School of Nursing, Fagin Hall, 418 Curie Blvd, Philadelphia, PA 19104 USA; 7 Greater Lawrence Family Health Center, 34 Haverhill Street, Lawrence, MA 01841 USA; 8 Department of Pharmacology, Bowles Center for Alcohol Studies, University of North Carolina, CB#7178, 104 Manning Road, Chapel Hill, NC 2759 USA; 9 University of North Carolina, 725 MLK Jr. Blvd., CB 7505, Chapel Hill, NC 27599 USA

**Correction : Harm Reduction Journal (2023) 20:1** **https://doi.org/10.1186/s12954-023-00879-7**

In the original version of this article [1], the paragraph under the section “Drug War Failure” should read as follows:

Xylazine is unscheduled at the federal level, and to date is illegal only in Florida, where it was banned in 2018 [40]. Increased attention to xylazine and its harms has recently prompted a call for xylazine to be federally scheduled. However, we urge caution in such an approach. Scheduling xylazine would only control the licit supply, creating barriers to needed research on the health impacts, toxicology, and safety profile of the drug. Further, xylazine is used very commonly as a surgical anesthetic for animals used in pre-clinical research, including cancer research. Scheduling xylazine would disrupt these research areas and slow progress.

The original article has been corrected.
